# Assessment of the Biocontrol Efficacy of Silver Nanoparticles Synthesized by *Trichoderma asperellum* Against Infected *Hordeum vulgare* L. Germination

**DOI:** 10.3390/life14121560

**Published:** 2024-11-27

**Authors:** Yasmin M. Heikal, Nada S. Shweqa, Hala M. Abdelmigid, Amal A. Alyamani, Hoda M. Soliman, Noura El-Ahmady El-Naggar

**Affiliations:** 1 Botany Department, Faculty of Science, Mansoura University, Mansoura 35516, Egypt; nadasalah2000@mans.edu.eg (N.S.S.); hudasoliman@mans.edu.eg (H.M.S.); 2 Department of Biotechnology, College of Science, Taif University, Taif 21944, Saudi Arabia; h.majed@tu.edu.sa (H.M.A.); a.yamani@tu.edu.sa (A.A.A.); 3 Department of Bioprocess Development, Genetic Engineering and Biotechnology Research Institute, City of Scientific Research and Technological Applications (SRTA-City), New Borg El Arab City 21934, Egypt; nelahmady@srtacity.sci.eg

**Keywords:** silver nanoparticles, biosynthesis, *Trichoderma asperellum*, statistical optimization, characterization, cyto-genotoxicity assay, biocontrol activity, barley

## Abstract

This study investigated the biosynthesis, statistical optimization, characterization, and biocontrol activity of silver nanoparticles (AgNPs) produced by newly isolated *Trichoderma* sp. The *Trichoderma asperellum* strain TA-3N was identified based on the ITS gene sequence, together with its phenotypic characteristics (GenBank accession number: OM321439). The color change from light yellow to brown after the incubation period indicates AgNPs biosynthesis. The UV spectrum revealed a single peak with the maximum absorption at 453 nm, indicating that *T. asperellum* produces AgNPs effectively. A Rotatable Central Composite Design (RCCD) was used to optimize the biosynthesis of AgNPs using the aqueous mycelial-free filtrate of *T. asperellum*. The optimal conditions for maximum AgNPs biosynthesis (156.02 µg/mL) were predicted theoretically using the desirability function tool and verified experimentally. The highest biosynthetic produced AgNPs by *T. asperellum* reached 160.3 µg/mL using AgNO_3_ concentration of 2 mM/mL, initial pH level of 6, incubation time of 60 h, and biomass weight of 6 g/100 mL water. SEM and TEM imaging revealed uniform spherical shape particles that varied in size between 8.17 and 17.74 nm. The synthesized AgNPs have a Zeta potential value of −9.51 mV. FTIR analysis provided insights into the surface composition of AgNPs, identifying various functional groups such as N–H, -OH, C-H, C=O, and the amide I bond in proteins. Cytotoxicity and genotoxicity assays demonstrated that AgNPs in combination with *T. asperellum* can mitigate the toxic effects of *Fusarium oxysporum* on barley. This intervention markedly enhanced cell division rates and decreased chromosomal irregularities. The results indicate that AgNPs synthesized by *T. asperellum* show the potential as an eco-friendly and efficient method for controlling plant diseases. Further studies are necessary to investigate their possible use in the agricultural sector.

## 1. Introduction

Nanotechnology is an emerging area that focuses on the design, characterization, and manufacturing of devices and systems by changing the shape and size to nanoscale [1,2]. Significant advances in nanotechnology have been recorded, as researchers have identified different ways to build and use nanostructured substances in a range of disciplines (e.g., biology, medicine, pharmacy, agriculture, food, textile, and electronics sectors) [3,4]. Conventional NP synthesis often involves hazardous reagents and complex procedures, posing environmental and safety risks. Consequently, researchers have investigated chemical, biological, and physical methods [5,6]. Novel synthesis protocols are essential for producing NPs with diverse compositions, shapes, sizes, and uniformity to advance nanotechnology [7,8]. Metal NPs can be synthesized utilizing metals in either top-down or bottom-up approaches [9]. Almost any metal can be introduced in the form of NPs [10]. The most employed metals for NP synthesis include silver (Ag), gold (Au), iron (Fe), aluminum (Al), copper (Cu), cobalt (Co), zinc (Zn), and cadmium (Cd) [11]. However, green synthesis is a viable alternative that is cost-effective, environmentally benign, sustainable and single-step process. Plant extracts, algae, bacteria, fungi, and other microbes are an excellent and benign source for AgNPs synthesis [7]. They play a crucial role as a reducing, capping, and stabilizing agent to facilitate the synthesis of AgNPs. The biological synthesis of AgNPs involves utilizing extracellular enzymes secreted by fungi [12,13], facilitating nanoparticle creation, nanostructure formation, and biomimetic mineralization [14,15]. Hence, fungi have significantly contributed to sustainable nanomaterial production [16].

*Trichoderma asperellum* (family Hypocreaceae) is an important agricultural biocontrol agent, producing enzymes that reduce metal ions, making it suitable for synthesizing AgNPs. Various *Trichoderma* species efficiently synthesize green NPs using diverse extracellular enzymes [17,18]. These fungi combat soil pathogens, such as *Rhizoctonia solani*, *Pythium ultimum*, and *Fusarium* spp., significantly damaging crops. *Trichoderma virens* (GL-21) controls damping-off disease in U.S. vegetable transplants and greenhouses [19], whereas *Trichoderma stromaticum* is used commercially to manage broom disease in *Amazonian cacao* [20]. Studies have shown that *Trichoderma harzianum* and *Ganoderma sessile* promote plant development and inhibit detrimental fungi in important crops [21].

AgNPs are an environmentally sustainable alternative to chemical fungicides for the protection of plants against *F. oxysporum* [22,23]. Applications of nanotechnology in agriculture offer advantages such as prolonged shelf life, enhanced pesticide solubility, reduced toxicity, and targeted uptake [24,25]. To enhance the practicality and economic viability of AgNPs for real-world applications, various approaches have been explored. These involve optimizing production techniques for efficient and sustainable synthesis, enhancing application methods with precise delivery systems and controlled-release formulations, exploring synergistic effects by combining AgNPs with other biological control agents, conducting cost-benefit analyses, mitigating environmental and safety risks through research and regulations, examining multifunctional applications for pest management and plant growth, and collaborating with industry for technology transfer, field testing, and developing user-friendly formulations. These endeavors aim to establish AgNPs as a viable option for sustainable agriculture and plant disease management [26].

This study is directed to optimize AgNPs production using *Trichoderma asperellum* and assess its efficacy against plant fungal and bacterial diseases, thereby promoting sustainable disease management and advancing green nanotechnology. However, scaling up AgNPs production with *Trichoderma* faces challenges, such as achieving consistent results under different environmental conditions and affecting synthesis efficiency. Thus, it is necessary to identify the optimal conditions for the maximum AgNPs yield and quality.

Industrial production requires modification of bioreactor designs to enhance mixing and oxygen transfer for *Trichoderma* growth and enzyme production. Large-scale NPs purification and characterization face challenges including effective residue elimination and consistent particle sizing for quality [27]. Design of experiments (DOE) is preferred over methods such as build-test-fix [28] and one-factor-at-a-time (OFAT) [29,30] due to its systematic analysis of complex systems, efficient input-output relationship exploration, reduced experimental runs, and identification of critical interactions [31,32]. Rotatable central composite design (RCCD) effectively optimizes physicochemical parameters [33,34] and identifies key factors and interactions affecting AgNPs formation to determine optimal conditions for synthesizing AgNPs with desired attributes that are essential for scientific applications [35].

Compliance with toxicity assessments and regulations is crucial for the safe production of AgNPs. Cost-effective synthesis optimizes factors that improve the shelf life, stability, and bioactivity [36]. Thus, a multidisciplinary approach is required to achieve scalable and sustainable production. Evaluating the genetic impact of AgNPs in agriculture is crucial, as they can damage cellular DNA, leading to mutations, cancer, and other health issues [37]. Genotoxicity is a key aspect of this safety evaluation. It is critical to determine suspected mutagens and/or cancer-causing agents through the identification of primary DNA lesions, chromosomal damage, or recombination and gene mutations [38]. The assessment of genotoxicity is vital before widespread agricultural use. Cytogenotoxicity tests measure the impact of AgNPs on plant cell DNA to identify potential hazards. Chromosomal irregularities and changes in the mitotic cycles across plant species indicate environmental genotoxicity [39,40,41,42]. Previous studies have shown that AgNPs and metal oxide NPs can cause genetic damage [43]. AgNPs notably increase chromosomal abnormalities and micronuclei [44]. High concentrations of nCeO_2_ slightly decrease the mitotic index, suggesting potential genotoxicity, whereas nTiO_2_ NPs have no effect [45]. Both AgNPs and AgNO_3_ exhibit cytotoxicity and genotoxicity at concentrations ranging from 1.00 to 1.75 µg/mL, as measured by the micronucleus assay [46]. Further studies are necessary to fully understand the genotoxic potential of nanoparticles.

*Fusarium* infections are widespread and are mostly associated with plant diseases. They are a major source of economic concern in agriculture because of agricultural losses caused by mycotoxin contamination of cereal grains. Most *Fusarium* species may trigger a wide range of plant diseases that damage numerous crops, including major agricultural and commercial crops like wheat, barley, maize, bananas, and cotton, often with disastrous socioeconomic consequences. Barley (*Hordeum vulgare* L.) is one of the cereal crops which are an important food source for humans and animals, but they are frequently attacked by fungal infections [47]. *Trichoderma* spp.-mediated AgNPs synthesis offers a sustainable, eco-friendly, and cost-effective approach to enhance crop yield and quality while reducing reliance on synthetic pesticides. This method demonstrates potent antimicrobial effects, mitigates environmental contamination, and improves soil health by promoting plant growth and microbial activity. The accessibility of this approach to small-scale farmers and their integration into Integrated Pest Management (IPM) strategies contribute to enhanced crop health [48].

This study aimed to enhance green nanotechnology by optimizing AgNPs biosynthesis using *Trichoderma asperellum*. We focused on improving the production efficiency and consistency and evaluating AgNPs’ biocontrol efficacy against plant pathogens. A Rotatable Central Composite Design (RCCD) investigated four variables: incubation time, pH, AgNO_3_ concentration, and biomass weight. AgNPs were characterized and tested against *Fusarium oxysporum*. Additionally, cytological and genotoxicity assays were conducted to assess the effects of AgNPs on chromosomal stability and DNA integrity in barely cells. This study offers insights into the potential applications of AgNPs in sustainable agriculture for disease management and crop protection.

## 2. Materials and Methods

### 2.1. Fungal Biomass Preparation

*Trichoderma* sp. was obtained from the Mycology Laboratory, Botany Department, Mansoura University, Egypt. Potato Dextrose Agar (PDA) medium was prepared with 200 g potato extract, 20 g dextrose, and 20 g agar per liter of distilled water, autoclaved at 12 °C for 20 min, and poured into Petri dishes. The fungal isolate was incubated on PDA at 25 °C for five days to ensure optimal growth, then stored at 4 °C for future applications.

### 2.2. Microscopic Characterization of the Fungal Isolate

The vegetative and reproductive structures of the fungal isolate were examined under a digital light microscope at magnifications of 100×.

*Trichoderma* sp. strain TA-3N was cultured on PDA plates and incubated at 25 °C for five days. The gold-coated dehydrated fungal growth specimen was examined at different magnifications using SEM (JSM-6510 L.V, JEOL Ltd., Tokyo, Japan) at EM Unit, Mansoura University, Egypt [49].

### 2.3. Molecular Identification of Fungal Isolate

Fungal DNA was extracted using a modified method of Panabieres et al. [50]. PCR amplification of the ITS region of the rDNA was performed using primers ITS1 (5′-TCC GTA GGT GAA CCT GCG G-3′) and ITS4 (5′-TCC TCC GCT TAT TGA TAT GC-3′), designed with Primer3 software version 4.1.0 (https://primer3.org/, accessed on 1 July 2024) and synthesized by BIONEER Inc., Oakland, CA, USA. PCR amplification was performed using a standard program, and the amplified DNA was visualized by gel electrophoresis on a 2% agarose gel with ethidium bromide (0.5 μg/mL) and documented using a MicroDoc system (Cleaver Scientific Ltd., Rugby, Warwickshire, UK). Purified PCR products were sequenced on an ABI 3730 XL DNA Analyzer (Applied Biosystems, Carlsbad, CA, USA), and the sequence was subjected to a BLASTn search against the NCBI GenBank database (http://blast.ncbi.nlm.nih.gov/, accessed on 15 July 2024) for identification. Phylogenetic analysis was conducted using the neighbor-joining method [51] and the phylogenetic tree was created using MEGA 11 software [52]. Sequence data were deposited in the NCBI GenBank database.

### 2.4. Extracellular Synthesis of AgNPs

*T. asperellum* strain TA-3N was grown on a PDA medium, and three disks were transferred to flasks containing PDA broth. After incubation at 25 ± 1 °C and 150 rpm for 72 h, the fungal mass was filtered and washed. Subsequently, 10 g of fungal fresh-weight was suspended in 100 mL sterile deionized water and shaken at 150 rpm and 25 ± 1 °C for 24 h. AgNO_3_ (1 mM) was added to the aqueous mycelial-free filtrate, and the flasks were incubated in shaker incubator in the dark at 30 °C and 150 rpm for 72 h. Flasks without silver ions were prepared for the control treatment. AgNPs production is indicated by the color change to dark brown after the incubation period of AgNO_3_ and the aqueous mycelial-free filtrate mixture. In contrast, the absence of AgNO_3_ was not associated with any color change. The maximum absorbance of the produced green synthesized AgNPs was determined within the 300–1000 nm wavelength range using UV-Vis spectroscopy.

### 2.5. Optimization of Biosynthesis Parameters

Rotatable Central Composite Design (RCCD) was utilized to optimize AgNP biosynthesis with *Trichoderma asperellum*. The examined variables were AgNO_3_ concentration (X_1_), initial pH level (X_2_), incubation time (X_3_), and biomass weight (X_4_). Each of these factors was evaluated at five distinct levels, represented as −2, −1, 0, 1, and 2. The design comprised 30 runs, including 6 center-point runs. This design identifies significant factors, their optimal values, and evaluates factors interactions. Experimental data from RCCD were analyzed using a second-order polynomial equation to understand the relationship between independent variables (e.g., AgNO_3_ concentration, pH, incubation time, biomass weight) and the response variable (AgNPs yield). The results were fitted to the following second-order polynomial equation:

$$Y=\beta_{0}+\sum_{i}\beta_{i}X_{i}+\sum_{ii}\beta_{ii}X_{i}^{2}+\;\sum_{ij}\beta_{ij}\;X_{i}\;X_{j}$$

where *Y* is the predicted response variable (AgNPs yield), X_1_, X_2_, X_3_, and X_4_ are the independent variables. $X_{i}$ and Xj are the coded levels of independent variables which are coded as X_1_, X_2_, X_3,_ and X_4_, $\beta_{ij}$ is the interaction coefficient, $\beta_{i}$ is the linear coefficient, $\beta_{ii}$ is the quadratic coefficient, and ${\;\beta }_{0}$ is the regression coefficient [53].

### 2.6. Characterization of the Biosynthesized AgNPs

The physical and chemical characteristics of biosynthesized AgNPs were evaluated using several analytical techniques. AgNP fabrication was confirmed using UV-Vis absorption spectroscopy (Unicam UV-VIS Spectrometer UV2, USA), which recorded the absorption spectrum between 200 and 1000 nm using deionized water as a reference [54].

To conduct Scanning Electron Microscopy (SEM), the centrifuged AgNP suspension was dropped onto a glass surface and dehydrated. The analysis was conducted using low-vacuum SEM (JEOL JSM-6510 L.V, JEOL Ltd., Tokyo, Japan) at 30 kV. The morphology and size of the AgNPs were examined using Transmission Electron Microscopy (TEM). The sample, dried on a carbon-coated grid (Type G 200, 3.05 µm diameter, EMS, Hatfeld, PA, USA), was analyzed with a JEOL JEM 2100, Tokyo, Japan [55].

Energy-dispersive X-ray Analysis (EDX) was employed to confirm the existence of elemental silver. This analysis was conducted using an X-ray micro-analyzer (Oxford 6587 INCA, Oxford Instruments, Abingdon, UK), which was linked to a JEOL JSM-6510 L.V scanning electron microscope. The microscope was operated at 20 kV [56].

The stability of the solution was evaluated utilizing a Zeta Potential Analyzer (Zetasizer ver. 7.01, Malvern Instruments, Westborough, MA, USA). The analysis required the addition of 5 mg of AgNPs to 5 mL of ultrapure water, after which the mixture was stirred at ambient temperature for 30 min.

Fourier Transform Infrared Spectroscopy (FTIR) analyzed surface chemistry by detecting functional groups through infrared absorption frequencies from 400 to 4000 cm^−1^, with a resolution of 4 cm^−1^. AgNPs were dispersed in a dry KBr matrix, formed AgNPs-KBr disk, and analyzed using a JASCO FTIR (Tokyo, Japan) spectrometer.

### 2.7. Biocontrol of Seed Rot Disease Caused by F. oxysporum Using AgNPs Biosynthesized by Trichoderma asperellum

#### Germination and Morphological Assessment of Barley Seeds

*Hordeum vulgare* L. (barley) seeds were sourced from the Agriculture Research Center (of) in Giza. The seeds were surface sterilized with 0.01% HgCl_2_ for 60 s, rinsed with distilled water, dried on filter paper, and exposed to various AgNPs concentrations (0, 10, 20, and 30 mg/L) and *Fusarium oxysporum* spores for 24 h. Post-treatment, seeds were placed on sterilized Whatman filter paper in Petri dishes and incubated for 7 days, with daily observations to monitor germination and morphological changes.

The effect of AgNPs on barley seeds was examined by evaluating several germination indicators. The initial metric assessed was Germination percentage (G%), which provided insights into how AgNPs affected seed viability [57]. Germination indices such as the final germination percentage (FGP), peak value (PV), mean daily germination (MDG), and germination value (GV) were calculated using methods from [58]. The mean germination time (MGT) was determined following Ellis and Roberts [59]. Seed mortality (SM) was calculated based on that described by Orchard [60], Osman [61], Lin et al. [62], and Mekki et al. [63].

To evaluate the initial growth characteristics in barley seedlings, a random selection of twelve seedlings was made from each experimental group. Measurements were recorded for shoot length (SL), root length (RL), and entire seedling length (SEL). The inhibition percentages for shoots (SI), roots (RI), and whole seedlings (SEI) were determined by comparing the lengths of treated seedlings to control seedlings. The method described by Lin et al. [62] was employed to assess the fresh weight (FW), dry weight (DW), and water content (WC). Following the methods described by Rogers et al. [64], several weight ratios were measured, including those for root, shoot, shoot-to-root, and root-to-shoot. The seedling vigor indices (SVI and SVII) were determined employing the methodology proposed by Abdul-Baki and Anderson [65].

To calculate the percentage of root and shoot inhibition, the following formulas were used:

Root inhibition percentage: (RLc/RLt) × 100

Shoot inhibition percentage: (SLc/SLt) × 100

Seedling inhibition percentage: (Seedling length of control/Seedling length of treatment) × 100

where RLc is the root length of the control, RLt is the root length of the treatment, SLc is the shoot length of the control, and SLt is the shoot length of the treatment. The results were recorded as the mean of triplicates ± standard error (S.E).

### 2.8. Cytological Preparations and Genotoxicity Assay

Barley seeds (*Hordeum vulgare* L.) were germinated on moist filter paper at 26 °C, and the root tips were subjected to various concentrations of AgNPs and *F. oxysporum* spores to evaluate their impact. Two control groups were used: distilled water (T_1_) and *F. oxysporum* spores alone (T_2_). Other treatments included a 1 mM AgNO_3_ solution (T_3_), and AgNPs at concentrations of 10 mg/L (T_4_), 20 mg/L (T_5_), and 30 mg/L (T_6_). Additional treatments combined AgNPs with *F. oxysporum* spores: 10 mg/L AgNPs (T_7_), 20 mg/L AgNPs (T_8_), and 30 mg/L AgNPs (T_9_).

Root tips (0.5–1 cm) were collected from seedlings, fixed in Carnoy’s solution, hydrolyzed in 1 N HCl (60 °C, 3 min), and stained with Carbol Fuchsin and Aceto-Orcein [66]. Specimens were dried, coversliped, gently warmed, and excess fluid removed to achieve uniform stain distribution. Researchers used an Olympus CX31RTSF Model microscope (Olympus, Tokyo, Japan) at 1000× magnification with oil immersion to examine at least 2000 cells from approximately 10 slides per treatment for chromosomal irregularities. Aberrant cells were quantified, and representative images were digitally captured (Toup Cam X Full HD camera, Hangzhou, China) and processed (Photoshop 8.0). Calculations for the mitotic index, phase indices, and total abnormality percentage were performed by enumerating various mitotic stages and examining chromosomal irregularities. These calculations were used to assess the genotoxic effects of AgNPs on barley cells. The formulas for these calculations are as follows:

(1) $$Mitotic\;index\;\left(MI\right)=\frac{TDC}{TC}\times 100$$

(2) $$Phase\;index\;\left(PI\right)=\frac{TP}{TDC}\times 100$$

(3) $$Total\;percentage\;of\;abnormal\;cells=\frac{Tabn}{TDC}\times 100$$

where TDC represents the total number of cells undergoing division, TC denotes the overall count of cells examined, Tabn signifies the sum of cells exhibiting abnormalities, and TP indicates the cell number observed in each specific phase.

### 2.9. Statistical Analysis

Statistical analysis of the results was conducted using SPSS (version 22.0, 2013, IBM Corp., Armonk, NY, USA), employing one-way analysis of variance (ANOVA) to identify statistically significant differences among the control and treated groups. For post hoc comparisons, Tukey’s multiple range test was applied (*p* ≤ 0.05) [66]. Design-Expert software (Version 12, Stat-Ease, Inc., Minneapolis, MN, USA) was utilized to create the optimization design. The experimental data were subjected to multiple regression analysis to calculate the analysis of variance (ANOVA), *p*-value, *F*-value, and confidence levels. Additionally, the coefficient of determination (R^2^) and adjusted R^2^ were determined. To generate three-dimensional surface plots, STATISTICA software (Version 8, StatSoft, Inc., Tulsa, OK, USA) was employed.

## 3. Results

### 3.1. Fungal Identification

**Figure 1:**
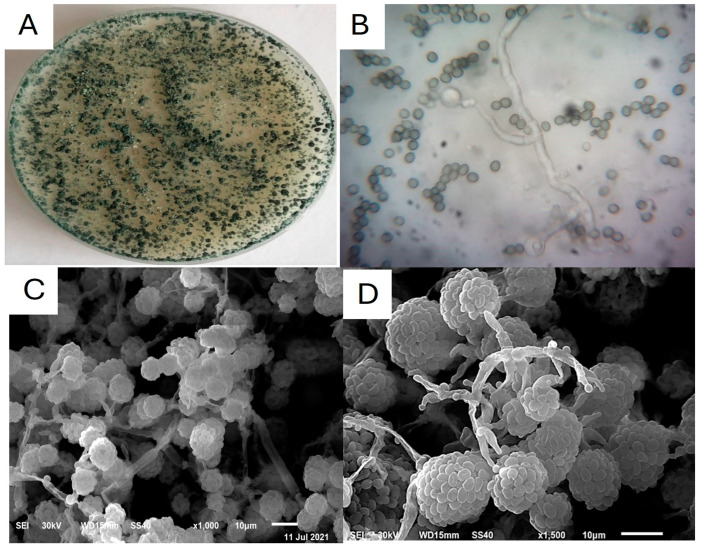
Morphological and structural identification of *Trichoderma* sp. strain TA-3N: (**A**) characteristic growth of *Trichoderma* sp. strain TA-3N on PDA medium after 7 days of incubation at 25 °C; (**B**) microscopic features displaying septate and branched mycelium along with conidia observed by light microscopy at 100×; and (**C**,**D**) scanning electron microscopy (SEM) images.

The identification of *Trichoderma* sp. strain TA-3N was conducted in this study utilizing both morphological characteristics and molecular analysis. Morphological investigation demonstrated the presence of green mycelia on culture plates (Figure 1A). A light microscope was used to investigate mycelia and spores’ morphology (Figure 1B), where branched mycelium with septate hyphae was observed. Conidia was identified as oval to round and green in color. The scanning electron micrograph was used to study the conidia surface (Figure 1C,D). The conidia of *Trichoderma* sp. strain TA-3N are oval or slightly globose. A group of conidia arranged together to resemble a ball. The conidiophores are branched and the phialides are pear-shaped.

**Figure 2:**
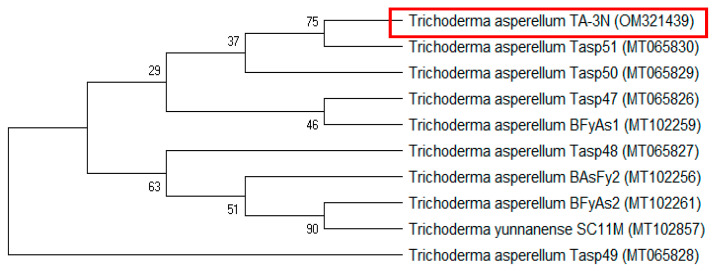
Phylogenetic tree of *Trichoderma* sp. strain TA-3N: This phylogenetic tree was constructed using a sequence from ITS regions of *Trichoderma* sp. strain TA-3N and closely related species. The tree was constructed utilizing 1000 bootstrap replicates, with accession numbers for the sequences indicated in parentheses.

The phylogenetic analysis of *Trichoderma* sp. strain TA-3N is illustrated in Figure 2. Genomic DNA was isolated from the fungal mycelia, followed by amplification, purification, and sequencing of the ITS gene region using the designated primers. The obtained sequence of *Trichoderma* sp. strain TA-3N (321 bp) was deposited under accession number OM321439 within the GenBank database and compared with fungal sequences in the GenBank database using BLAST. The obtained sequence was confirmed by BLAST to be 100% similar to the ITS sequences of *Trichoderma asperellum*.

Phylogenetic analysis (Figure 2) revealed two distinct groups: a larger cluster comprising nine isolates, including *T. asperellum* TA-3N (OM321439) and its close relative *T. asperellum* Tasp51 (MT065830), and a smaller cluster consisting solely of *T. asperellum* Tasp49 (MT065828). Phylogenetic analysis verified that *Trichoderma* sp. strain TA-3N was closely related to *Trichoderma asperellum*, which confirmed the identification based on morphological characteristics. Based on the analysis of the ITS gene sequence and its phenotypic features, *Trichoderma* sp. strain TA-3N was identified as *Trichoderma asperellum* isolate TA-3N.

### 3.2. Biosynthesis and Characterization of AgNPs

**Figure 3:**
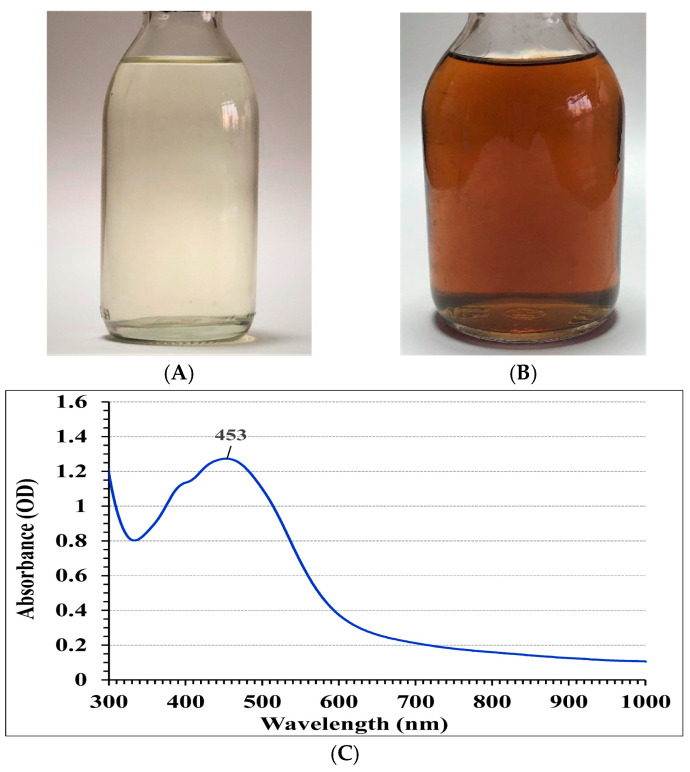
The color change demonstrates the biosynthesis of AgNPs using *T. asperellum*. Bottle (**A**) represents the control (aqueous mycelial-free filtrate without AgNO_3_); Bottle (**B**) shows the test flask (aqueous mycelial-free filtrate with AgNO_3_) after 72 h of incubation. (**C**) displays the UV-Vis spectral scan (ranging from 300 to 1000 nm) of the biosynthesized AgNPs.

AgNPs were successfully produced through biosynthesis by *T. asperellum*. The resulting AgNPs were analyzed using UV-Vis spectroscopy, as shown in Figure 3A–C. The observed color transition from light yellow to brown following the incubation period indicates the occurrence of the synthesis process. Examination of the ultraviolet-visible spectrum, spanning from 300 to 1000 nm, showed a single peak with the highest absorption at 453 nm, validating the effective biosynthesis of AgNPs by *T. asperellum*.

### 3.3. Optimization of Biosynthesis Parameters Using RCCD

The optimization of AgNPs biosynthesis using the aqueous mycelial-free filtrate of *T. asperellum* was conducted using a Rotatable Central Composite Design (RCCD). Four process variables were examined: the AgNO_3_ concentration (X_1_), initial pH level (X_2_), incubation time (X_3_), and biomass weight (X_4_). The lowest AgNPs yield (20.79 µg/mL) was obtained in run No. 15 using 2 mM/mL silver nitrate concentration, pH 8, 36 h incubation, and 6 g biomass per 100 mL water. Conversely, the highest AgNPs yield of 158.80 µg/mL was attained in run No. 15 using 2 mM/mL AgNO_3_ concentration, pH 6, 60 h incubation period, and 6 g biomass per 100 mL water. A comparison of the experimental and predicted results for the biosynthesized AgNPs is presented in Table 1. The experimental and predicted results demonstrate a high degree of concordance, as shown in Table 1.

#### 3.3.1. Model Fit and Optimization

Analysis of variance (ANOVA) was conducted to assess the model’s fit and significance of the coefficients. The coefficient of determination (R^2^) was 0.9956, indicating a strong fit. The adjusted R^2^ was 0.9916, and the predicted R^2^ was 0.9783. The model’s *F*-value of 244.21, and a *p*-value below 0.0001, indicated a strong statistical significance of the model (Table 2). Linear coefficients for AgNO_3_ concentration (X_1_), incubation time (X_3_), and biomass weight (X_4_) showed *p*-values less than 0.0001, whereas the initial pH level (X_2_) had a *p*-value of 0.0721. These results suggest that all variables, except the initial pH level, significantly influenced AgNPs biosynthesis. Regarding the quadratic coefficients, X_1_ was not statistically significant (*p*-values of 0.9353), whereas X_2_, X_3_, and X_4_ were statistically significant (*p* < 0.05). The quadratic impact of the initial pH level, incubation time, and biomass weight on AgNPs biosynthesis was demonstrated by *F*-values of 14.12, 203.13, and 1186.77, respectively. Significant interaction effects were observed between the AgNO_3_ concentration and incubation time (*p*-value < 0.0001), between the initial pH level and incubation time (*p*-value = 0.0121), between the initial pH level and biomass weight (*p*-value < 0.0001), and between the incubation time and biomass weight (*p*-value < 0.0001). In contrast, the interaction effects between AgNO_3_ concentration and initial pH level, and between AgNO_3_ concentration and initial pH level were not significant.

Analysis of the coefficients revealed factors that positively influence AgNPs biosynthesis (Table 2). The linear effects of AgNO_3_ concentration (X_1_) and incubation time (X_3_) were found to positively influence the biosynthesis of AgNPs. Positive interactions were identified between AgNO_3_ concentration and initial pH level (X_1_X_2_), AgNO_3_ concentration and incubation time (X_1_X_3_), AgNO_3_ concentration and biomass weight (X_1_X_4_), and initial pH and biomass quantity (X_2_X_4_). While negative interactions were identified between the initial pH level and incubation time (X_2_X_3_), and the incubation time and biomass weight (X_3_X_4_). Quadratic effects of AgNO_3_ concentration (X_1_), incubation time (X_3_), and biomass weight (X_4_) were found to positively influence the biosynthesis of AgNPs. The positive coefficients indicate synergistic relationships among the interacting factors, with their crucial role in enhancing AgNPs biosynthesis, when indicated by the significant *p*-values (<0.0001). In addition, the current model’s adequate precision value of 55.47 indicated its capability to navigate the design space.

Table 3 displays the RCCD fit summary analysis for AgNPs biosynthesis using *T. asperellum* strain TA−3 N. This analysis was used to determine the most appropriate model for AgNP biosynthesis among the linear, two-factor interactions (2FI), and quadratic models. The selection process considered the significance of the model terms and the non-significance of the lack-of-fit tests. The fit summary results revealed that the quadratic model was the most appropriate for silver nanoparticles biosynthesis, with significant *p*-value of < 0.0001 and a non-significant lack-of-fit (with *F*-value of 2.26 and *p*-value of 0.19) (Table 3). The quadratic model also exhibited the minimum standard deviation value (3.5), maximum R^2^ (0.9956), adjusted R^2^ (0.9916), and predicted R^2^ (0.9783).

To examine the correlation between the independent and dependent variables, a second-order polynomial equation was employed to determine the optimal values of AgNO_3_ concentration (X_1_), initial pH (X_2_), incubation time (X_3_), and biomass weight (X_4_) for maximum AgNPs biosynthesis. The following second-order polynomial equation can predict AgNPs biosynthesis (Y) based on the independent variables (X_1_, X_2_, X_3_, and X_4_):

Y = 47.78 + 4.38 X_1_ − 1.38 X_2_ + 16.75 X_3_ − 4.11X_4_ + 0.49 X_1_X_2_ + 11.04 X_1_X_3_ + 1.61X_1_X_4_ − 2.50 X_2_X_3_ + 28.33 X_2_X_4_ − 11.71X_3_X_4_ + 0.06 X_1_^2^ − 2.51X_2_^2^ + 9.54 X_3_^2^ + 23.05 X_4_^2^ (4)

where Y represents the predicted AgNP biosynthesis, X_1_ denotes the AgNO_3_ concentration value, X_2_ represents the initial pH level value, X_3_ represents the incubation time value, and X_4_ denotes the biomass weight.

#### 3.3.2. Three-Dimensional (3D) Surface Plots

**Figure 4:**
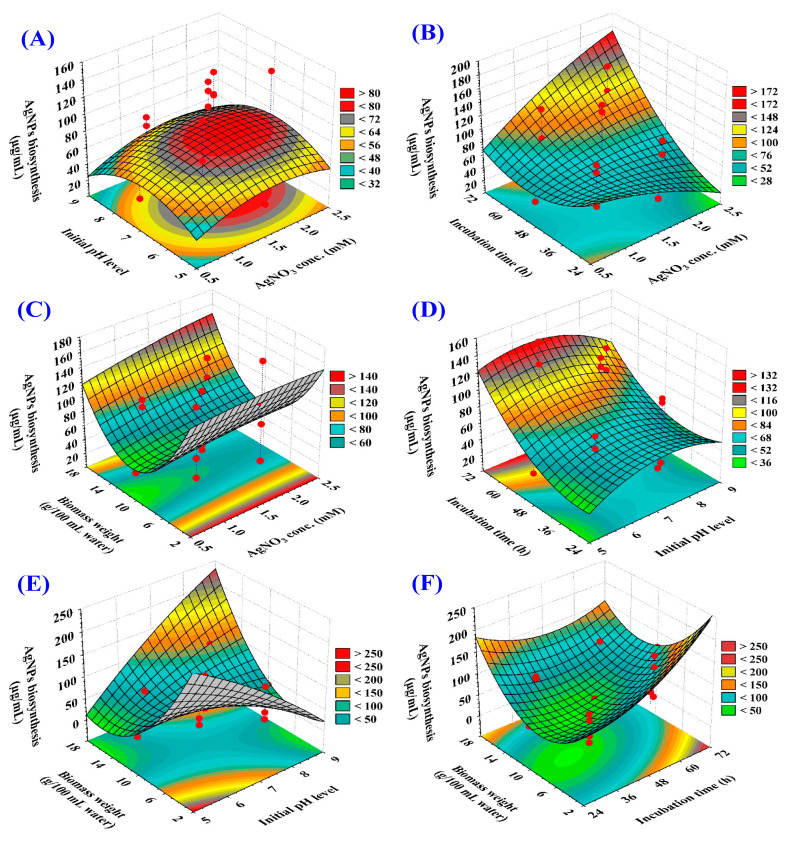
Three-dimensional plots showing the mutual effects of AgNO_3_ conc. (X_1_), initial pH level (X_2_), incubation time (X_3_), and biomass weight (X_4_) on AgNPs biosynthesis by *T. asperellum.* (**A**–**C**) show the effect of AgNO_3_ concentration on AgNPs biosynthesis when interacting with initial pH level, incubation period and biomass weight; respectively. (**A**,**D**,**E**) show the effect of initial pH level on AgNPs biosynthesis when interacting with AgNO_3_ concentration, incubation time, and biomass weight; respectively. (**B**,**D**,**F**) show the effect of incubation time on AgNPs biosynthesis when interacting with the AgNO_3_ concentration, initial pH level and biomass weight; respectively. (**C**,**E**,**F**) show the effect of biomass weight on AgNPs biosynthesis when interacting with the AgNO_3_ concentration, initial pH level and incubation time; respectively.

The three-dimensional response surface graphs identified the optimal variable levels for maximizing AgNPs biosynthesis and explored the interactions between the independent variables, including initial pH, incubation time, AgNO_3_ concentration, and temperature (Figure 4). Three-dimensional surface plots were generated by plotting AgNPs production (µg/mL) on the *z*-axis against two independent parameters, while the other two variables were held at their central levels.

Three-dimensional surface plots (Figure 4A–C) depict the interactive effects of AgNO_3_ concentration with the initial pH level, incubation time, and biomass weight on AgNPs biosynthesis. The plots show that AgNPs biosynthesis increased with increasing AgNO_3_ concentration and the highest AgNPs yield was attained by using 2 mM/mL AgNO_3_ concentration.

Figure 4A,D,E shows 3D response surface plots illustrating how the initial pH influences AgNPs biosynthesis when interacting with AgNO_3_ concentration, incubation time, and biomass weight. According to the plots, the optimal biosynthesis at an initial pH of approximately six, further increases in the initial pH level reduced AgNPs biosynthesis process.

Figure 4B,D,F presents 3D response surface plots illustrating the interactive effects among the incubation time and AgNO_3_ concentration, the initial pH level, and biomass weight on AgNPs biosynthesis. Longer incubation time enhanced AgNPs biosynthesis, with optimal biosynthesis at about 60 h incubation period.

Three-dimensional surface plots (Figure 4C,E,F) depict the interactive effects among the biomass weight and AgNO_3_ concentration, the initial pH level, and incubation time on AgNPs biosynthesis. The results showed that the highest AgNPs biosynthesis was achieved at a low level of the biomass weight (attained by using 6 g biomass per 100 mL water), further increase in the biomass weight level reduced the AgNPs biosynthesis process.

#### 3.3.3. The Model’s Adequacy

**Figure 5:**
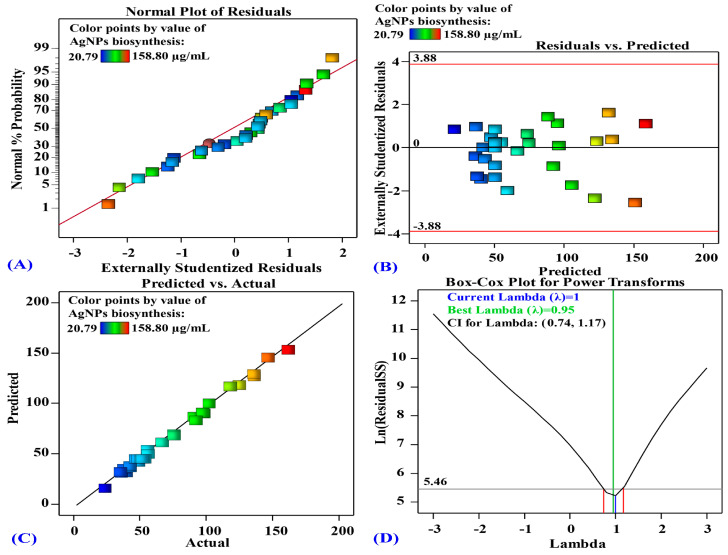
(**A**) Normal probability plot of internally studentized residuals. (**B**) A plot of internally studentized residuals versus predicted values. (**C**) Plot of predicted versus actual. (**D**) Box–Cox plot of model transformation of silver nanoparticle biosynthesis by *T. asperellum* as affected by AgNO_3_ conc. (X_1_), initial pH level (X_2_), incubation time (X_3_), and biomass weight (X_4_).

Figure 5 illustrates the normal probability plot of residuals, with data points aligning closely to a straight line, indicating that the residuals follow a normal distribution. Residuals represent the differences between the actual AgNPs biosynthesis values obtained through experiments and those calculated using the theoretical model. The model predictions closely matched the experimental data, demonstrating the high accuracy of the model. The plot of residuals against predicted values for the biosynthesis of silver nanoparticles is shown in Figure 5B. This graph demonstrates that the residuals were scattered randomly around the zero line. The graph in Figure 5C illustrates the correlation between the predicted and actual values of AgNPs biosynthesis. The distribution of data points along the diagonal line indicated strong agreement between the theoretical predictions of the model and the experimental results of AgNPs biosynthesis. The Box–Cox plot of the model transformation for AgNPs biosynthesis is shown in Figure 5D. In Figure 5D, the optimal lambda value (λ = 0.95) is depicted by a green line, while the blue line indicates the current Lambda (λ = 1). The red lines denote the minimum and maximum values of the 95% confidence interval, which were calculated as 0.74 and 1.17, respectively. The model was considered optimal as the current Lambda’s blue line fell between the two vertical red lines.

#### 3.3.4. Desirability Function

**Figure 6:**
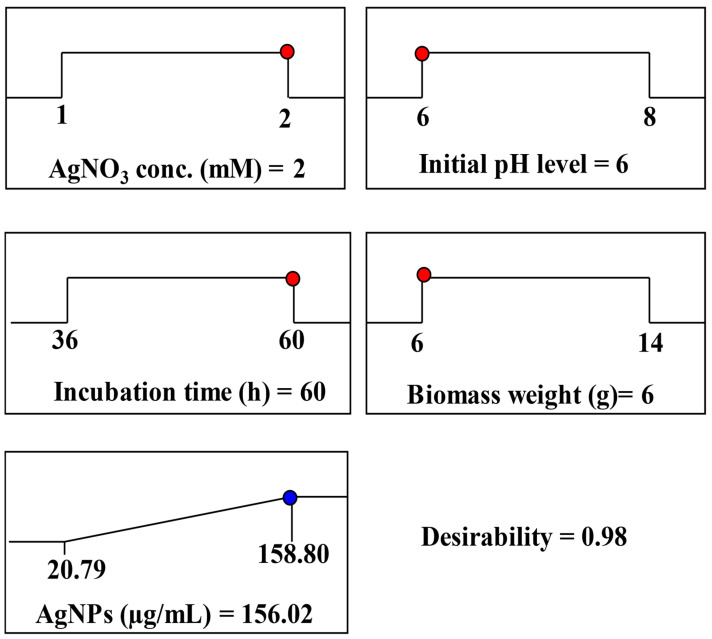
The optimization plot displayed the desirability function and the optimum predicted values of AgNP biosynthesis by *T. asperellum*.

The desirability function tool in the Software Design Expert (version 12) was used to calculate the optimal predicted conditions for maximum AgNPs biosynthesis by *T. asperellum* as affected by AgNO_3_ conc. (X_1_), initial pH level (X_2_), incubation time (X_3_), and biomass weight (X_4_) (Figure 6).

The optimum predicted conditions for the maximum AgNPs biosynthesis (156.02 µg/mL) were as follows: incubation time of 60 h, initial pH level of 6, AgNO_3_ concentration of 2 mM/mL, and biomass quantity of 6 g. An experiment was conducted under the optimal conditions predicted by DF to verify the results and validate the model’s accuracy. At optimal process variable levels, the maximal experimental biosynthesis of AgNPs by *T. asperellum* reached 160.3 µg/mL. The verification indicated that the experimental results and predicted values were closely correlated. A high degree of model accuracy is revealed by the verification, which indicates that the DF accurately predicts the levels of the process variables.

### 3.4. Characterization of the Biosynthesized AgNPs

**Figure 7:**
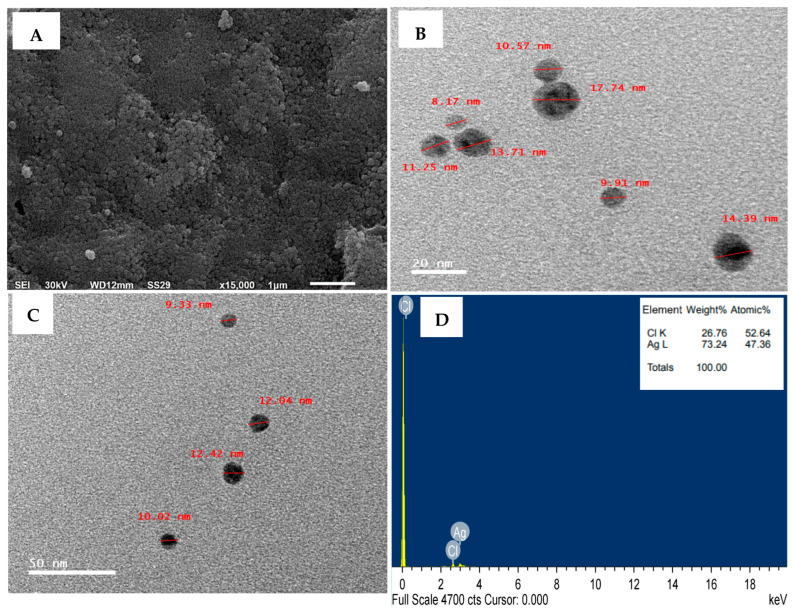
Biosynthesized AgNPs by *T. asperellum*: (**A**) scanning electron microscopy (SEM), (**B**,**C**) transmission electron microscopy (TEM) micrographs, and (**D**) energy-dispersive X-ray spectroscopy (EDX) analysis demonstrating the elemental composition of native silver.

This study utilized SEM, TEM, EDX, Zeta potential, and FTIR to assess the physical and morphological characteristics of AgNPs synthesized by *T. asperellum*. SEM analysis revealed spherical particles that were uniform and homogeneous (Figure 7A). TEM imaging (Figure 7B,C) revealed the morphology and size distribution of AgNPs, displaying individual particles that varied in size between 8.17 and 17.74 nm. Aggregation was prevented by a protein-based capping agent surrounding the nanoparticles, which created repulsive forces. Energy-dispersive X-ray spectroscopy (EDX) via TEM (Figure 7D) was used to investigate the types, distributions, and concentrations of elements in AgNPs. EDX spectrum confirmed the presence of Ag as the main component. Structural changes in AgNPs may occur during fabrication, and EDX can detect such variations.

**Figure 8:**
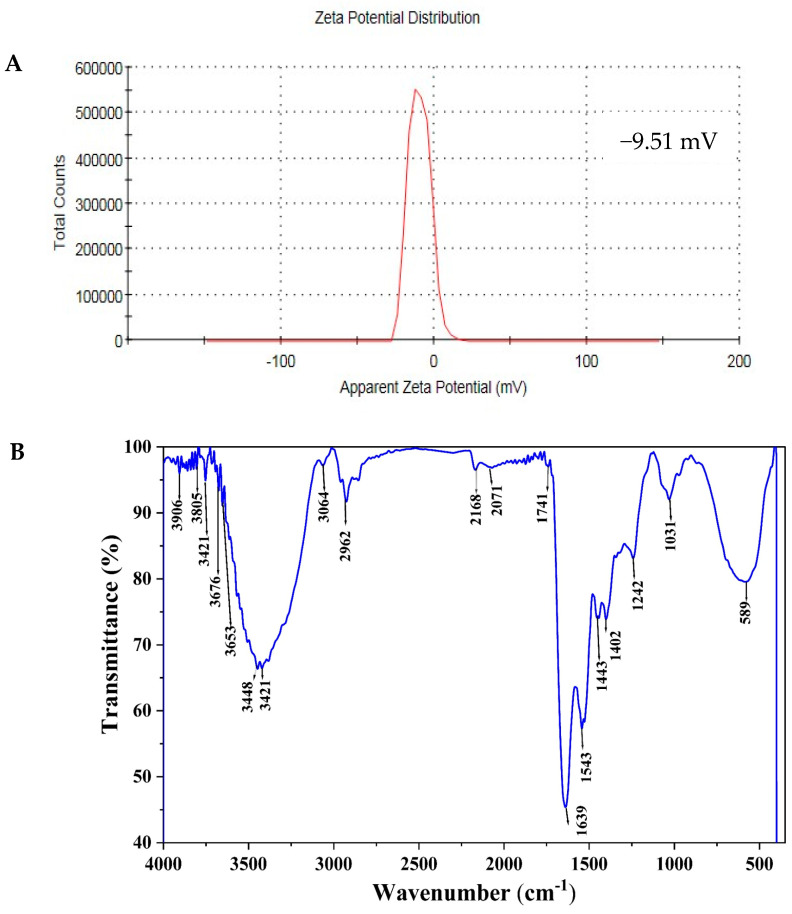
AgNPs synthesized by *T. asperellum* exhibit (**A**) Zeta potential and (**B**) Fourier transform infrared (FTIR) spectrum of various functional groups responsible for stabilizing or capping AgNPs.

The synthesized AgNPs have a Zeta potential value of −9.51 mV, which is represented by a single peak, as shown in Figure 8A. High negative or positive Zeta potentials in suspensions cause particles to repel each other, thereby preventing aggregation.

FTIR analysis identified functional groups stabilizing and reducing AgNPs. The FTIR spectrum for AgNPs synthesized using *T. asperellum* showed peaks at 3448, 3064, 2926, 1741, 1639, 1031, and 589 cm^−1^ (Figure 8B). Peaks at 3448 and 3064 cm^−1^ correspond to N–H stretching of primary and secondary amides and O–H stretching of alcohols, phenols, and carboxylic acids. The 2926 cm^−1^ peak indicates C–H symmetrical stretching of alkanes. The 1741 cm^−1^ peak is associated with C=O stretching in carboxylic acids, while the 1639 cm^−1^ peak reflects the carbonyl stretch of the amide I bond in proteins. The 1031 cm^−1^ peak indicates aliphatic amines, and the 589 cm^−1^ peak signifies C–Cl stretching in alkyl halides.

### 3.5. Biocontrol of Barley Seed Rot Disease Using T. asperellum-Synthesized AgNPs

#### 3.5.1. Germination and Morphological Assessment of Barley

**Figure 9:**
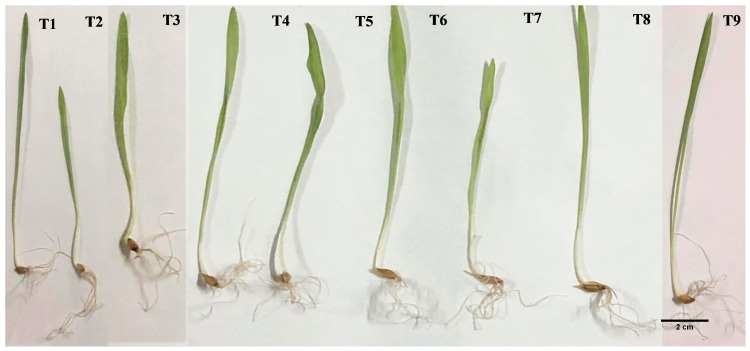
Seedling germination traits of *H. vulgare* under different concentrations of AgNPs, *F. oxysporum* treatment, silver nitrate, and a combination of these treatments. Abbreviations: T_1_, Dist water (control); T_2_, spore suspension of *F. oxysporum*; T_3_, silver nitrate solution 1 mM; T_4_, (10 mg/L of AgNPs); T_5_, (20 mg/L of AgNPs); T_6_, (30 mg/L of AgNPs); T_7_, 10 mg/L of AgNPs and spore suspension of *F. oxysporum*; T_8_, 20 mg/L of AgNPs and spore suspension of *F. oxysporum*; T_9_, (30 mg/L of AgNPs and spore suspension of *F. oxysporum*).

Barley seeds were immersed for 24 h in nine treatments: distilled water (control, T_1_); *Fusarium oxysporum* spore suspension (T_2_); 1 mM AgNO_3_ solution (T_3_), 10, 20, and 30 mg/L AgNPs (T_4_–T_6_); and combinations of these AgNPs concentrations with *F. oxysporum* spore suspension (T_7_–T_9_). The seeds were then germinated in triplicate for 5 days (Figure 9). The use of *F. oxysporum*, AgNO_3_, AgNPs, and their combinations significantly impacted various germination metrics, including the final germination percentage (FGP), mean germination time (MGT), mean daily germination (MDG), peak value (PV), germination value (GV), and seed mortality rate (SM). Table 4 represents the mean ± SE of three measurements relative to the control. T_5_ exhibited the highest FGP, MDG, and GV values (94.44%, 2.27% d^−1^, and 43.11% d^−2^, respectively), followed by T_7_ (91.67%, 2.20% d^−1^, and 40.33% d^−2^), significantly outperforming T_2_, which had the lowest values (19.45%, 0.47% d^−1^, and 1.89% d^−2^). MGT was substantially increased in T_4_ (1.50 day) and T_7_ (1.24 day), while T_8_ showed the lowest value (1.12 day). Notable PV increases were observed in T_5_ (18.80% d^−1^) and T_7_ (18.33% d^−1^), with T_2_ having the lowest value (3.89% d^−1^) compared to the control (18.89% d^−1^). The results indicated that T_2_ had the highest SM value (193.33%) while T_5_ had the lowest (13.33%), similar to the control (13.33%).

Various treatments involving *F. oxysporum*, AgNO_3_, AgNPs, and their combination significantly impacted the early growth parameters of barley seedlings, including shoot length (SL), root length (RL), seedling length (SEL), shoot inhibition (SI) (%), fresh weight (FW), dry weight (DW) (g), water content (WC) (%), root inhibition (RI) (%), seedling inhibition (SEI) (%), vigor index I (SVI), and vigor index II (SVII). The results, shown as the mean values of triplicates ± standard error (SE), are detailed in Table 5 and Table 6. For RL, T_6_ (6.50 cm) and T_9_ (5.75 cm) had the highest values, while T_2_ (4.50 cm) and T_7_ (4.00 cm) had the lowest, compared to the control (5.75 cm). T_6_ (13.50 and 20.00 cm) and T_9_ (12.50 and 18.25 cm) showed significant increases in SL and SEL, whereas T_2_ (9.75 and 14.25 cm) showed a notable decrease compared to the control (11.25 and 17.00 cm). Regarding the fresh weight (FW), T_6_ showed a notable increase of 2.94 g, while T_3_, T_5_, T_7_, and T_9_ exhibited significant decreases of 1.96 g, 1.95 g, 1.94 g, and 1.95 g, respectively, in contrast to the 3.08 g observed in the control group. Regarding the DW, treatments T_6_ and T_7_ yielded the highest values at 0.35 g and 0.36 g, respectively. Conversely, T_2_ (0.25 g), T_4_ (0.24 g), and T_9_ (0.26 g) produced the lowest results when compared to the control group, which measured 0.27 g (Table 5).

The highest RI values were observed in T_2_ (1.30%) and T_7_ (1.44%), while T_3_ and T_6_ exhibited the lowest values (0.89% each) in comparison to the control. Additionally, T_2_ exhibited a significant increase in SI and SEI, showing values of 1.15 and 1.20%, respectively, when compared to the control. In contrast, T_6_ demonstrated a notable reduction, with values of 0.84 and 0.85%. The SVI measurements were considerably higher for T_5_ (1637.50) and T_7_ (1581.25), while T_7_ displayed the highest SVII value at 32.54. In contrast, T_2_ (300.00 and 5.25) significantly decreased both SVI and SVII compared to the control (1558.3 and 24.75). Regarding water content percentage, T_2_ exhibited the maximum value at 91.39%, whereas T_7_ showed the minimum at 81.68%, in comparison to the control group’s 91.20% (Table 6).

#### 3.5.2. Cytogenetic Evaluation of *T. asperellum*-Synthesized AgNPs in Barley

##### Mitotic Activity

**Figure 10:**
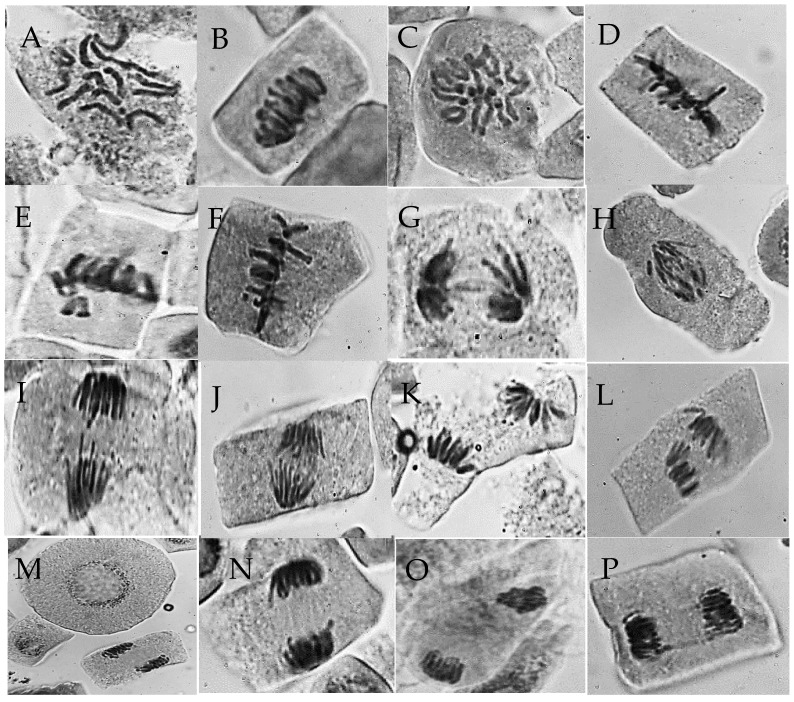
Various chromosome aberrations observed in *H. vulgare* under different treatments for 24 h: (**A**) diploid cell metaphase (2n =14) after (T_2_), (**B**) stickiness metaphase after (T_1_), (**C**) ring at metaphase after (T_2_), (**D**) disturbed metaphase after (T_5_), (**E**) large non-congression at metaphase after (T8), (**F**) small non-congression at metaphase after (T_9_), (**G**) bridge at anaphase after (T_3_), (**H**) laggard anaphase after T_9_, (**I**) late separation anaphase after T_9_, (**J**) oblique anaphase after T_2_, (**K**) diagonal anaphase after T_4_, (**L**) disturbed anaphase after T_4_, (**M**) oblique telophase and abnormal cell size after T_5_, (**N**) late separation telophase after T_2_, (**O**) diagonal telophase after T_1_, and (**P**) bridge telophase after T_2_ with magnification 1000× except M at 400×.

The impact of varying AgNPs and *F. oxysporum* spore concentrations on barley (*Hordeum vulgare*) root tips was investigated over 24 h, focusing on the effects on prophase, metaphase, anaphase, and telophase mitotic stages (Table 7 and Table 8, Figure 10). The study also assessed the frequency and nature of chromosomal abnormalities caused by these treatments, providing insights into their genotoxic potential on barley root cells. Our findings highlighted the diverse effects of the different treatments on the mitotic activity of barley root cells. The observed significant increase in MI for several treatments (T_3_, T_5_, T_7_, T_8_, and T_9_) suggests potential stimulatory effects on cell division, whereas the reduced MI values in T_2_ and T_4_ indicate possible inhibitory effects (Table 7).

Moreover, further analysis of the specific mitotic phases and chromosomal abnormalities could provide valuable insights into the mechanisms underlying these varied responses to AgNPs and *F. oxysporum*. As depicted in Table 7, analysis of the mitotic phases showed that T_5_ treatment had the highest prophase occurrence relative to the control. Additionally, treatments T_2_, T_3_, T_4_, T_5_, and T_7_ displayed a significant increase in metaphase frequency compared to the control. Among the samples analyzed, T_8_ demonstrated the greatest frequency of anaphase, followed by T_9_. T_6_ had the highest frequency of telophase, followed in descending order by T_7_, T_9_, T_2_, T_4_, T_5_, T_3_, and T_8_.

#### 3.5.3. Chromosomal Abnormalities

Table 7 and Figure 10 illustrate the results of genotoxicity testing to examine chromosomal abnormalities in mitotic cells of barley under various treatments. The highest frequency of abnormal mitosis was observed in T_2_ (23.68%), followed by T_8_, T_3_, T_6_, T_9_, T_5_, and T_7_, whereas the lowest frequency was observed in T_4_ (11.38%) compared to the control (7.76%). Table 8 illustrates the frequencies of various types of abnormal interphase and mitotic cells observed in each treatment. Treatment of *H. vulgare* caused diverse mitotic abnormalities, primarily in metaphase, including stickiness, non-congression, oblique metaphase, chromosome ring formation, and disturbance, with stickiness, non-congression, and disturbance being most frequent; however, no interphase aberrations were observed.

In the metaphase stage, chromosomes displayed adhesive properties, creating a homogeneous chromatin cluster. This phenomenon was most evident in T_2_ and least noticeable in T_9_. Chromosome non-congression, the failure to align at the equatorial plate, was frequent in T_2_, T_3_, and T_5_ but rare in T_7_ and T_8_. Oblique metaphase was most frequently observed in treatments T_5_, T_4_, and T_3_, with other treatments showing patterns similar to the control. The disturbed metaphase was the most common abnormality, particularly in T_8_, T_5_, and T_6_, and was least prevalent in T_7_. Chromosome ring formation was most obvious in T_3_ and T_2_, and least observed in T_9_, T_1_, and T_4_.

As depicted in Table 8, various chromosomal irregularities were identified during the anaphase and telophase, including delayed chromosome separation, diagonal orientation, bridge formation, oblique alignment, lagging chromosomes, and disrupted arrangements. Bridge formation was most frequent in T_3_ (anaphase) and T_2_/T_9_ (telophase), whereas delayed separation was particularly common in T_2_. T_9_ (anaphase) and T_6_ (telophase) exhibited the highest occurrence of lagging chromosomes. Diagonal orientation was most prevalent in T_6_/T_7_ (telophase) and T_4_/T_8_ (anaphase), whereas oblique alignment was most common in T_5_ (telophase) and T_4_ (anaphase). The disturbed configurations were most notable in T_9_ (anaphase) and T_3_ (telophase), with T_5_ (anaphase) and T_2_ (telophase) showing the lowest frequency. These observations underscore the substantial effect of these treatments on chromosomal stability during cell division.

## 4. Discussion

Nanotechnology enhances agricultural productivity by using a variety of delivery agents, including nanoherbicides, nanofungicides, nanofertilizers, nanopesticides for plant protection against pests, and nanosensors, to identify diseases in crops, monitor plant growth, conduct genetic engineering, and manage post-harvest production. Agro-nanobiotechnology is currently being found to be used in water management, target gene transference, nano-barcoding, agro-nanosensors, restricted agrichemical discharge, seed germination, phytohormone delivery, and more [67].

Fungi are known as a promising source for the biological synthesis of nanoparticles owing to their abundant protein content, high nanoparticle yield, simplicity of manipulation, and generation of little hazardous byproducts. In the synthesis of metal nanoparticles, fungi function as both stabilizing and reducing agents. Biomolecules found in fungi serve as a capping agent for nanoparticles preventing the over-growth of nanoparticles, enhancing their biological activity, shielding them to prevent agglomeration, and increasing their stability. Proteins and amino acids are considered the main biomolecules that function as capping agents. It is also presumed that the negative carboxyl groups that exist in the cell wall enzymes contribute to the electrostatic attraction between the nanoparticles and these biomolecules [68]. The presence of proteins on the surface of the nanoparticles for their stabilization was confirmed by Gudikandula et al. [69], who synthesized biogenic silver nanoparticles using two white-rot fungi. Biologically synthesized silver nanoparticles were reported to have signals for oxygen and carbon in EDS analysis, indicating the adsorption of organic molecules from the culture filtrate onto the nanoparticles [68].

A Rotatable Central Composite Design (RCCD) was applied in order to optimize the conditions for the biosynthesis of silver nanoparticles by *T. asperellum*. Multiple-regression statistical analysis and analysis of variance (ANOVA) were used to analyze the relationship between the independent factors and the biosynthesis of AgNPs by the aqueous mycelial-free filtrate of *T. asperellum* (Table 2). The model’s fit was confirmed using the determination coefficient (R^2^). Mohamedin et al. [70] indicate that R^2^ values reflect the degree to which experimental variables and their interactions explain the response variability. The R^2^ value of 0.9956 in the current investigation suggests that the model is fit and capable of explaining 99.56% of the variability in AgNPs biosynthesis by the aqueous mycelial-free filtrate of *T. asperellum*. Furthermore, the model is incapable of explaining just 0.44 percent of the total variability in the biosynthesis of AgNPs by *T. asperellum*. Higher R^2^ values, closer to one, denote a more accurate predictive design [71] and strong alignment between predicted and observed data [72]. R^2^ values exceeding 0.9 indicate a statistically significant correlation [73]. In addition, the adjusted determination coefficient (Adj. R^2^ = 0.9916) is also very high, confirming the model’s significance. The predicted-R^2^ is a metric that measures the model’s capacity to predict responses for new experiments. Consequently, our findings indicate that the model is capable of accurately predicting the value of AgNPs biosynthesis by the aqueous mycelial-free filtrate of *T. asperellum* with a precision of 0.9783 within the evaluated factors range. Additionally, the adjusted-R^2^ value of 0.9916 is in reasonable agreement with the predicted-R^2^ value of 0.9783 for AgNPs biosynthesis by the aqueous mycelial-free filtrate of *T. asperellum*, which confirms the model’s statistical validity and reliability. A model is considered highly significant and accurate when the predicted-R^2^ and adjusted-R^2^ values differ by less than 20% [74]. Process variables were considered to have significant effects if their *p*-values were less than or equal to 0.05 (confidence levels greater than or equal to 95 percent) [75,76].

In addition, the study investigated the coefficients’ signs to determine whether they had a positive or negative impact on the response [75]. The negative values of the linear, mutual interactions or quadratic coefficients indicate an antagonistic relationship between AgNPs biosynthesis by the aqueous mycelial-free filtrate of *T. asperellum* and the tested variables (negative effects of the factors on AgNPs biosynthesis by the aqueous mycelial-free filtrate of *T. asperellum*), whereas the positive linear, mutual interactions or quadratic coefficient values indicate a synergistic relationship between the variables and AgNPs biosynthesis by the aqueous mycelial-free filtrate of *T. asperellum* in the tested range of these variables.

The fit summary results contributed to selecting the proper model that fits the RCCD used for AgNPs biosynthesis by the aqueous mycelial-free filtrate of *T. asperellum*. The quadratic model with non-significant lack of fit, lower standard deviation, lower PRESS value, a very low *p*-value, higher R^2^ value, adjusted R^2^ value, and predicted R^2^ value is the adequate model fitting AgNPs biosynthesis by the aqueous mycelial-free filtrate of *T. asperellum*.

Three-dimensional response surface graphs were used to determine the optimal levels and interactions of independent variables (initial pH, incubation time, AgNO_3_ concentration, and biomass weight) for maximizing AgNPs biosynthesis by *T. asperellum*. Process parameters, including temperature, incubation time, initial pH, and reactant concentration, significantly influence nanoparticle size [77]. It is well established that pH significantly affects the size, shape, and stability of AgNPs [78].

The normal probability plot (NPP) of the residuals is an essential graphical technique for determining the adequacy of the model and visualizing the residuals’ distribution. The residuals are the difference between the experimental response values and the predicted response values of the theoretical model. A small residual value indicates that the model’s prediction is very precise and that the experimental results are well-fitted by the model. The residual points are normally distributed and are situated adjacent to the diagonal straight line, which suggests the model’s validity. A plot of the predicted AgNPs biosynthesized by the aqueous mycelial-free filtrate of *T. asperellum* vs. the studentized residuals is shown in Figure 5B. The residuals exhibited a uniform and random distribution around the zero line, without any obvious pattern, indicating constant variance and verifying the model‘s precision [79]. Figure 5C illustrates a plot of predicted versus actual AgNPs biosynthesized by the aqueous mycelial-free filtrate of *T. asperellum*. The data points clustered along the fitted line confirm a strong correlation between the model‘s predictions and the experimental results of AgNPs biosynthesis by the aqueous mycelial-free filtrate of *T. asperellum* and confirm the accuracy of the model [80]. The Box–Cox plot for AgNPs biosynthesis using *T. asperellum* indicated that the current Lambda value (λ = 1) fell within the optimal range, suggesting that the model adequately explained the experimental results without requiring data transformation [81].

A desirability function (zero being undesirable and one desirable) was employed to determine optimal parameters for maximum response values [82,83]. The study identified the best conditions for AgNPs biosynthesis by the aqueous mycelial-free filtrate of *T. asperellum* as a 60 h incubation period, initial pH of 6, 2 mM/mL AgNO_3_ concentration, and 6 g biomass. Under these conditions, the maximum predicted AgNPs yield was 156.02 µg/mL.

Many analytical techniques, including UV-visible spectroscopy, TEM, SEM, EDX, Zeta potential, and FTIR, were employed to characterize AgNPs synthesized by *T. asperellum*. In accordance with earlier research [5,84,85], these techniques accomplished a comprehensive analysis of the structural and physical characteristics of NPs. The UV-visible spectrum of biosynthesized AgNPs showed a surface plasmon resonance (SPR) absorption band at 453 nm, indicating the collective oscillation of free electrons in the AgNPs upon light interaction. This SPR band is consistent with previous reports on AgNPs, typically found between 300 and 500 nm [86]. SEM and TEM analyses revealed spherical particles ranging from 8.17 to 17.74 nm, with some clumping observed. These findings align with earlier studies on AgNPs synthesized using *T. virens* [87], and the nanoparticle sizes fall within the typical 1–50 nm range [88]. EDX analysis determined the elemental composition of the biosynthesized AgNPs, revealing a predominant silver signal (73.24% by weight) and a minor chloride peak (26.76%). The chloride presence likely results from biomolecules adhering to the AgNPs’ surface [89].

The Zeta potential measurements of AgNPs showed a value of −9.51 mV, indicating repulsive forces between NPs, confirmed by a single peak. High absolute Zeta potential values prevent aggregation, while low values can cause flocculation [90]. FTIR analysis showed peaks at 3448, 3064, 2926, 1741, 1639, 1031, and 589 cm^−1^. The 1639 cm^−1^ peak was related to primary amine stretching vibrations [91] and amide I and II bending vibrations in the proteins [92]. The band at 3448 cm^−1^ corresponded to primary amine stretching vibrations [93]. FTIR confirmed that phenols and proteins act as reducing, stabilizing, and capping agents for AgNPs, aiding the conversion of silver radicals to ions [94,95].

*Trichoderma* species are efficacious biological control agents against plant-pathogenic fungi, promoting growth and mitigating *Fusarium* wilt [96]. These organisms effectively manage soil-borne pathogens, such as *Rhizoctonia solani*, *Pythium ultimum*, and *Fusarium* spp., which are causative agents of damping-off disease in crops. For instance, *Trichoderma virens* (GL-21) controls damping-off in US vegetable seedlings and greenhouse plants [19]. Mycogenic AgNPs synthesized using biocontrol agents exhibit inhibitory effects on various plant disease-causing fungi. *Trichoderma* exhibits exceptional efficacy owing to its diverse mechanisms, including mycoparasitism through cell wall-degrading enzymes, production of antibiotic secondary metabolites, competition for resources and territory, induction of systemic immune responses in plants, and bioremediation of deleterious soil substances [17,18]. These mechanisms enable *Trichoderma* to exert control over pathogens and enhance plant development. This study investigated the extracellular production of AgNPs by *T. asperellum* and their efficacy against barley seed rot diseases. Zaki et al. [97] demonstrated that *Trichoderma harzianum*-synthesized AgNPs efficiently and eco-friendly manage fungal pathogens in crops, particularly Egyptian cotton (*Gossypium barbadense* L.). At the molecular and cellular levels, AgNPs suppress *F. oxysporum* spore germination by disrupting physiological processes, compromising cell membrane integrity, and increasing cellular permeability. This leads to intracellular component leakage caused by reactive oxygen species (ROS) and free radicals, resulting in cell wall damage and lipid peroxidation. Additionally, AgNPs interfere with carbohydrate, amino acid, and energy metabolism, inhibiting mycelial growth and conidial germination, as well as causing protein denaturation and nucleic acid degradation [97]. Upcoming research will explore these mechanisms to elucidate the biocontrol effectiveness of AgNPs produced by *Trichoderma asperellum* in combating *F. oxysporum*.

Previous studies have indicated that silver nano-solutions can improve seed germination and suppress fungal infections. Karimi et al. [98] discovered that seeds coated with silver nano-solution germinated more successfully than those treated with conventional fungicides. Yasur and Rani [99] showed that even high concentrations of AgNPs did not adversely affect the germination or development of *Ricinus communis*. Previous studies have demonstrated that various nanomaterials such as TiO_2_ and SiO_2_NPs can improve seed germination rates. For instance, the application of TiO_2_NPs using electrospray techniques has been found to boost germination in both lettuce and wheat crops [100,101]. Similarly, SiO_2_NPs have provided a notable increase in seed germination in tomato plants [102]. In our study, the results proved that the combination of NPs with fungi (T_7_) significantly enhanced germination compared to the control. This observation suggests that the NPs may have attenuated the toxic effects of *Fusarium oxysporum* on barley. *F. oxysporum* exhibits a notable capacity to withstand and mitigate the adverse effects of heavy metals in contaminated environments. This fungus employs diverse mechanisms to neutralize heavy metals, including active efflux from its cellular structure, immobilization on cell walls to prevent deleterious effects, sequestration both extracellularly and intracellularly through complex formation with proteins and other compounds, and the synthesis of metallothioneins, which are proteins crucial for binding and detoxifying heavy metals. These defense mechanisms enable *F. oxysporum* to counteract the negative impacts of heavy metals on barley roots, thereby promoting root development and overall plant vigor [103].

The results of our research demonstrate that incorporating AgNPs into treatments resulted in significant enhancements in both shoot and root elongation, consistent with the observations reported by Emamverdian et al. [104]. Additionally, AgNPs were found to increase the fresh weight (FW), dry weight (DW), and water content (WC) in plants. When applied in combination with *F. oxysporum*, AgNPs mitigated the detrimental effects of the fungus on barley, suggesting a synergistic relationship. These findings support the notion that the effects of AgNPs on plants are primarily influenced by their concentration, specific plant species under investigation, and method of application [105]. Elamawi and Al-Harbi Kasem et al. [106], found that lower doses of AgNPs increased barley seed germination while decreasing the prevalence of *Fusarium oxysporum*-induced barley seed rot disease. However, greater levels of AgNPs inhibited barley grain germination and significantly reduced root length. The chlorosis of leaves was caused by the loss of chlorophyll pigments and the disorganization of chloroplast thylakoids in positive silver ions, as well as the treatment of barley groups with AgNPs. Thus, AgNPs provide a wide range of alternatives for tackling barley diseases.

Barley (*Hordeum vulgare* L.) serves as an ideal organism for investigating induced chromosomal abnormalities because of its small number (2n = 2x = 14) of comparatively large chromosomes. Researchers have extensively utilized domesticated barley as a model organism to investigate the effects of various physical and chemical agents on chromosomes. Chromosome and chromatid abnormalities have been detected in both somatic (root and shoot tips) and reproductive (microsporocytes) cells, demonstrating these effects [107]. This study examined *H. vulgare* root tips subjected to nine different treatments for 24 h. The treatments included distilled water, AgNO_3_ solution, *F. oxysporum* spore suspension, various concentrations of AgNPs, and combinations of AgNPs with *F. oxysporum* spore suspensions. Calculations were performed to determine how various treatments affect the mitotic indices (MI%), and phase indices (PI%), as well as the types and total percentage of abnormalities (Tab%).

Cytotoxicity can be assessed by examining the reduction in the mitotic index rate [44]. Changes in the mitotic index provide essential insights into the impact of potential toxins on cellular processes. By analyzing the rate of cell division, the effects of various compounds on cell division and tissue viability can be evaluated. This method quantitatively screens compounds to determine their harmful effects on cellular functions. Growth can be assessed using the mitotic index, which quantifies cells undergoing division. A lower mitotic index indicates diminished growth rates. In this study, the results of the cytotoxicity test revealed that AgNPs reduced the detrimental effects of *F. oxysporum*. Among all treatments, T_5_, T_7_, and T_9_ showed the highest MI%, while T_2_ demonstrated the lowest value. On the other hand, T_2_ showed the highest percentage of total abnormalities (Tab%), while T_4_ and T_7_ demonstrated the lowest. These results are consistent with earlier work conducted by Panda et al. [108], which demonstrated an increase in chromosomal abnormalities in *Allium cepa* cells exposed to AgNPs derived from *Pandanus odorifer*.

Within the same context, this study identified various chromosomal irregularities, including stickiness, non-congression, oblique metaphase, chromosome ring formation, disturbed metaphase, delayed separation, diagonal arrangement, bridge formation, oblique positioning, lagging chromosomes, and disrupted anaphase and telophase. These abnormalities, indicative of DNA damage, can significantly affect cellular division and genomic stability, leading to unequal genetic content distribution during mitosis or meiosis. Consequently, offspring cells may exhibit altered chromosome quantity or configuration, affecting cell functionality, organismal growth, and genetic stability. Irregularities stem from improper chromosome fiber folding, causing entanglement and aggregation [109]. Additionally, anaphase bridge formation may result from chromosomal fractures and reconnections [110]. The formation of cross-linkages between chromatin proteins can result in incomplete separation of daughter chromosomes, potentially leading to chromosome breakage and sticky anaphase [111]. Prior research has documented similar cytogenetic effects of AgNPs on *Allium cepa* and *Vicia faba* root tips [44,108]. Studies by Kumari et al. [112,113] showed chromosomal abnormalities in *Allium cepa* cells exposed to high AgNPs levels. Liman [114] also observed DNA damage and micronucleated cells in *Allium cepa* exposed to ZnO and bismuth (III) oxide nanoparticles. Current research suggests that *T. asperellum* may reduce AgNPs-induced genotoxicity in barley root tips, as indicated by fewer chromosomal abnormalities compared to the control treatments. These findings are consistent with those of Kasem et al. [106], who showed that *Trichoderma viride* mitigated the adverse effects of *F. oxysporum* on mitotic indices and chromosomal abnormalities in *Allium cepa*.

## 5. Conclusions

This research successfully demonstrated an efficient, economically viable, and environmentally sustainable method for the biosynthesis of AgNPs utilizing *T. asperellum*. The extracellular filtrate of the fungus verified the capability to synthesize AgNPs from silver nitrate solution. The synthesized AgNPs exhibited potential biocontrol activity against fungi. These findings indicate that AgNPs generated by *T. asperellum* may serve as an effective and eco-friendly solution for sustainable control of plant diseases. AgNPs help to reduce the prevalence of barley diseases, seed germination, and enhance the barley grain physiology. Further research is required to understand the long-term effects of these AgNPs on soil microbiota and plant health. Additionally, investigating the potential synergistic effects with other biocontrol agents and optimizing production and application methods could enhance the efficacy and cost-effectiveness of AgNPs for large-scale agricultural use. Future studies should also explore the molecular and cellular mechanisms of action to provide a comprehensive understanding of AgNPs biosynthesis and its applications in plant protection.

## Figures and Tables

**Table 1:** The Rotatable Central Composite Design matrix demonstrates the influence of AgNO_3_ concentration (X_1_), initial pH level (X_2_), incubation time (X_3_), and biomass weight (X_4_) on AgNPs biosynthesis using the aqueous mycelial-free filtrate of *T. asperellum*.

Std	Run	Variables				AgNPs (µg/mL)				Residuals	
		X_1_	X_2_	X_3_	X_4_	Experimental	Predicted				
20	1	0	2	0	0	32.27	34.95			−2.68	
26	2	0	0	0	0	49.12	47.78			1.34	
14	3	1	−1	1	1	72.70	70.92			1.78	
8	4	1	1	1	−1	95.39	92.57			2.82	
24	5	0	0	0	2	132.95	131.76			1.19	
18	6	2	0	0	0	52.86	56.76			−3.90	
23	7	0	0	0	−2	143.45	148.21			−4.76	
30	8	0	0	0	0	48.97	47.78			1.19	
12	9	1	1	−1	1	94.18	93.64			0.54	
28	10	0	0	0	0	45.54	47.78			−2.24	
16	11	1	1	1	1	121.79	120.79			1.00	
10	12	1	−1	−1	1	33.16	33.77			−0.61	
19	13	0	−2	0	0	39.60	40.49			−0.89	
27	14	0	0	0	0	43.90	47.78			−3.88	
6	15	1	−1	1	−1	158.80	156.02			2.78	
13	16	−1	−1	1	1	34.94	37.84			−2.90	
29	17	0	0	0	0	48.22	47.78			0.45	
15	18	−1	1	1	1	89.16	85.76			3.41	
21	19	0	0	−2	0	53.33	52.43			0.90	
4	20	1	1	−1	−1	20.79	18.56			2.23	
2	21	1	−1	−1	−1	72.85	72.01			0.84	
17	22	−2	0	0	0	39.57	39.23			0.33	
7	23	−1	1	1	−1	63.90	63.96			−0.07	
9	24	−1	−1	−1	1	46.28	44.86			1.43	
1	25	−1	−1	−1	−1	87.85	89.52			−1.68	
22	26	0	0	2	0	114.95	119.42			−4.47	
5	27	−1	−1	1	−1	133.07	129.37			3.70	
11	28	−1	1	−1	1	99.31	102.77			−3.46	
3	29	−1	1	−1	−1	36.58	34.12			2.46	
25	30	0	0	0	0	50.91	47.78			3.13	
**Variable**						**Code**	**Coded and Actual Levels**				
							**−2**	**−1**	**0**	**1**	**2**
AgNO_3_ conc. (mM)						X_1_	0.5	1	1.5	2	2.5
Initial pH level						X_2_	5	6	7	8	9
Incubation time (h)						X_3_	24	36	48	60	72
Biomass weight (g/100 mL water)						X_4_	2	6	10	14	18

**Table 2:** Analysis of variance of RCCD for AgNPs biosynthesis using the aqueous mycelial-free filtrate of *T. asperellum* as affected by AgNO_3_ conc. (X_1_), initial pH level (X_2_), incubation time (X_3_), and biomass weight (X_4_).

Source of Variance		Coefficient Estimate	Sum of Squares	Degrees of Freedom	Mean Square	*F*-Value	*p*-Value
Model	Intercept	47.78	41,988.72	14	2999.19	244.21	<0.0001 *
Linear effect	X_1_	4.38	460.69	1	460.69	37.51	<0.0001 *
	X_2_	−1.38	45.97	1	45.97	3.74	0.0721
	X_3_	16.75	6732.70	1	6732.70	548.21	<0.0001 *
	X_4_	−4.11	405.84	1	405.84	33.05	<0.0001 *
Interaction effect	X_1_ X_2_	0.49	3.84	1	3.84	0.31	0.5845
	X_1_ X_3_	11.04	1950.37	1	1950.37	158.81	<0.0001 *
	X_1_ X_4_	1.61	41.31	1	41.31	3.36	0.0866
	X_2_ X_3_	−2.50	99.95	1	99.95	8.14	0.0121
	X_2_ X_4_	28.33	12,840.63	1	12,840.63	1045.56	<0.0001 *
	X_3_ X_4_	−11.71	2195.39	1	2195.39	178.76	<0.0001 *
Quadratic effect	X_1_^2^	0.06	0.08	1	0.08	0.01	0.9353
	X_2_^2^	−2.51	173.43	1	173.43	14.12	0.0019
	X_3_^2^	9.54	2494.66	1	2494.66	203.13	<0.0001 *
	X_4_^2^	23.05	14,574.87	1	14,574.87	1186.77	<0.0001 *
Error effect	Lack of Fit		150.90	10	15.09	2.26	0.1899
	Pure Error		33.32	5	6.66		
R^2^	0.9956						
Adj R^2^	0.9916						
Pred R^2^	0.9783						
Adeq Precision	55.47						

* Significant values, *F*: Fishers function, *p*: Level of significance.

**Table 3:** Fit summary of RCCD for AgNPs biosynthesis by *T. asperellum* influenced by AgNO_3_ concentration (X_1_), initial pH level (X_2_), incubation time (X_3_), and biomass weight (X_4_).

Fit Summary					
Source	Sequential *p*-Value	Lack of Fit *p*-Value		Adjusted R^2^	Predicted R^2^
Linear	0.268	<0.0001 *		0.0503	−0.2382
2FI	0.0267	<0.0001 *		0.3704	0.3178
Quadratic	<0.0001 *	0.1899		0.9916	0.9783
**Sequential Model Sum of Squares**					
**Source**	**Sum of Squares**	***df***	**Mean Square**	***F*-Value**	***p*-Value** ***P* rob**
Linear vs. Mean	7645.19	4	1911.3	1.38	0.268
2FI vs. Linear	17,131.5	6	2855.25	3.12	0.0267
Quadratic vs. 2FI	17,212.1	4	4303.01	350.38	<0.0001 *
**Lack of Fit Tests**					
**Source**	**Sum of Squares**	***df***	**Mean**	***F*-Value**	***p*-Value**
Linear	34,494.43	20	1724.72	258.82	0.00 *
2FI	17,362.95	14	1240.21	186.11	0.00 *
Quadratic	150.90	10	15.09	2.26	0.19
**Model Summary Statistics**					
**Source**	**Standard Deviation**	**R^2^**	**Adjusted R^2^**	**Predicted R^2^**	**PRESS**
Linear	37.16	0.1813	0.0503	−0.2382	52,216.98
2FI	30.26	0.5875	0.3704	0.3178	28,769.72
Quadratic	3.5	0.9956	0.9916	0.9783	917.15

* Significant values; *df*, degree of freedom; PRESS, sum of squares of prediction error; 2FI, two factors interaction.

**Table 4:** Seed germination characteristics of barley (*H. vulgare*) under varying concentrations of AgNPs, *F. oxysporum* treatment, AgNO_3_, and combinations of these treatments.

Treatment	FGP (%)	MGT (Days)	MDG (% d^−1^)	PV (% d^−1^)	GV (%d^−2^)	SM (%)
T_1_	94.45 ± 2.78 ^c^	1.55 ± 0.14 ^a^	2.27 ± 0.07 ^c^	18.89 ± 0.56 ^c^	42.89 ± 2.56 ^d^	13.33 ± 6.67 ^a^
T_2_	19.45 ± 2.78 ^a^	1.33 ± 0.17 ^a^	0.47 ± 0.07 ^a^	3.89 ± 0.56 ^a^	1.89 ± 0.56 ^a^	193.33 ± 6.67 ^c^
T_3_	88.89 ± 7.35 ^c^	1.29 ± 0.08 ^a^	2.13 ± 0.18 ^c^	17.78 ± 1.47 ^c^	38.44 ± 6.14 ^cd^	26.67 ± 17.64 ^a^
T_4_	63.89 ± 2.78 ^b^	1.50 ± 0.19 ^a^	1.53 ± 0.07 ^b^	12.78 ± 0.55 ^b^	19.66 ± 1.67 ^b^	86.67 ± 6.67 ^b^
T_5_	94.44 ± 5.56 ^c^	1.44 ± 0.10 ^a^	2.27 ± 0.13 ^c^	18.80 ± 1.11 ^c^	43.11 ± 4.89 ^d^	13.33 ± 13.33 ^a^
T_6_	66.67 ± 4.81 ^b^	1.29 ± 0.02 ^a^	1.60 ± 0.12 ^b^	13.33 ± 0.96 ^b^	21.55 ± 3.08 ^bc^	80.00 ± 11.55 ^b^
T_7_	91.67 ± 0.00 ^c^	1.24 ± 0.03 ^a^	2.20 ± 0.00 ^c^	18.33 ± 0.00 ^c^	40.33 ± 0.00 ^d^	20.00 ± 0.00 ^a^
T_8_	83.33 ± 4.81 ^b^	1.12 ± 0.08 ^a^	2.00 ± 0.12 ^bc^	16.67 ± 0.96 ^bc^	33.55 ± 3.85 ^bcd^	40.00 ± 11.55 ^ab^
T_9_	83.33 ± 4.81 ^b^	1.29 ± 0.09 ^a^	2.00 ± 0.12 ^bc^	16.67 ± 0.96 ^bc^	33.55 ± 3.85 ^bcd^	40.00 ± 11.55 ^ab^

FGP, final germination percentage; MGT, mean germination time; MDG, mean daily germination; PV, peak value; GV, germination value; SM, seed mortality. The results were recorded as means of triplicates ± Standard Error (S.E). Different superscript letters indicated significant differences (*p* ≤ 0.05) (Tukey’s test). Abbreviations: T_1_, Dist water (control); T_2_, spore suspension of *F. oxysporum*; T_3_, silver nitrate solution 1 mM; T_4_, (10 mg/L of AgNPs); T_5_, (20 mg/L of AgNPs); T_6_, (30 mg/L of AgNPs); T_7_, 10 mg/L of AgNPs and spore suspension of *F. oxysporum*); T_8_, 20 mg/L of AgNPs and spore suspension of *F. oxysporum*; T_9_, (30 mg/L of AgNPs and spore suspension of *F. oxysporum*).

**Table 5:** The effects of various treatments involving different concentrations of AgNPs, *F. oxysporum* inoculation, AgNO_3_ application, and their interactive combinations on growth parameters of *H. vulgare* seedlings.

Treatment	Shoot Length (SL) (cm)	Root Length (RL) (cm)	Seedling Length (SEL) (cm)	Seedling Fresh Weight (FW) (g)	Seedling Dry Weight (DW) (g)	Water Content (WC) (%)
T_1_	11.25 ± 0.75 ^a^	5.75 ± 0.25 ^ab^	17.00 ± 1.00 ^a^	3.08 ± 0.08 ^b^	0.27 ± 0.02 ^a^	91.20 ± 0.87 ^de^
T_2_	9.75 ± 0.25 ^a^	4.50 ± 0.50 ^a^	14.25 ± 0.75 ^a^	2.91 ± 0.09 ^b^	0.25 ± 0.01 ^a^	91.39 ± 0.61 ^e^
T_3_	10.00 ± 0.50 ^a^	5.00 ± 0.00 ^ab^	16.50 ± 1.00 ^a^	1.96 ± 0.04 ^a^	0.32 ± 0.00 ^bc^	83.67 ± 0.34 ^ab^
T_4_	11.25 ± 2.75 ^a^	5.50 ± 0.00 ^ab^	16.75 ± 2.750 ^a^	2.04 ± 0.04 ^a^	0.24 ± 0.00 ^a^	88.23 ± 0.23 ^cd^
T_5_	12.10 ± 1.40 ^a^	5.60 ± 0.40 ^ab^	17.70 ± 1.800 ^a^	1.95 ± 0.05 ^a^	0.28 ± 0.00 ^ab^	85.63 ± 0.37 ^bc^
T_6_	13.50 ± 1.50 ^a^	6.50 ± 0.50 ^b^	20.00 ± 2.00 ^a^	2.94 ± 0.06 ^b^	0.35 ± 0.01 ^c^	88.09 ± 0.59 ^c^
T_7_	13.25 ± 0.75 ^a^	4.00 ± 0.00 ^a^	17.25 ± 0.75 ^a^	1.94 ± 0.06 ^a^	0.36 ± 0.01 ^c^	81.68 ± 0.83 ^a^
T_8_	11.50 ± 3.00 ^a^	5.50 ± 0.50 ^ab^	17.00 ± 3.50 ^a^	2.05 ± 0.05 ^a^	0.33 ± 0.01 ^bc^	84.15 ± 0.15 ^ab^
T_9_	12.50 ± 0.50 ^a^	5.75 ± 0.25 ^ab^	18.25 ± 0.75 ^a^	1.95 ± 0.05 ^a^	0.26 ± 0.00 ^a^	86.66 ± 0.34 ^bc^

The results were recorded as the mean of triplicates ± Standard Error (SE). Different superscript letters indicated the significant differences (*p* ≤ 0.05) (Tukey’s test). Abbreviations: T_1_, Dist water (control); T_2_, spore suspension of *F. oxysporum*; T_3_, silver nitrate solution 1 mM; T_4_, (10 mg/L of AgNPs); T_5_, (20 mg/L of AgNPs); T_6_, (30 mg/L of AgNPs); T_7_, (10 mg/L of AgNPs and spore suspension of *F. oxysporum*); T_8_, (20 mg/L of AgNPs and spore suspension of *F. oxysporum*); T_9_, (30 mg/L of AgNPs and spore suspension of *F. oxysporum*).

**Table 6:** Seedling germination characteristics, including shoot and root elongation inhibition and vigor indices I and II, of barley (*H. vulgare*) seedlings.

Treatment	Shoot Inhibition (SI) %	Root Inhibition (RI) %	Seedling Inhibition (SEI) %	Seedling Vigor Index I (SVI)	Seedling Vigor Index II (SVII)
T_1_	0	0	0	1558.3 ± 91.66 ^b^	24.75 ± 1.83 ^bcd^
T_2_	1.15 ± 0.105 ^b^	1.30 ± 0.200 ^ab^	1.20 ± 0.13 ^b^	300.00 ± 75.00 ^a^	5.25 ± 1.25 ^a^
T_3_	1.12 ± 0.015 ^b^	0.89 ± 0.03 ^ab^	1.03 ± 0.00 ^b^	1366.66 ± 54.16 ^b^	26.66 ± 2.66 ^cd^
T_4_	1.05 ± 0.190 ^b^	1.04 ± 0.04 ^ab^	1.03 ± 0.110 ^b^	1035.41 ± 102.08 ^ab^	15.00 ± 1.00 ^ab^
T_5_	0.94 ± 0.045 ^b^	1.03 ± 0.03 ^ab^	0.96 ± 0.045 ^b^	1637.50 ± 312.50 ^b^	25.66 ± 2.33 ^cd^
T_6_	0.84 ± 0.040 ^b^	0.89 ± 0.03 ^a^	0.85 ± 0.035 ^b^	1241.66 ± 41.66 ^ab^	21.91 ± 2.08 ^bc^
T_7_	0.85 ± 0.010 ^b^	1.44 ± 0.06 ^b^	0.99 ± 0.015 ^b^	1581.25 ± 68.75 ^b^	32.54 ± 0.46 ^d^
T_8_	1.03 ± 0.205 ^b^	1.05 ± 0.05 ^ab^	1.03 ± 0.155 ^b^	1502.08 ± 377.08 ^b^	28.41 ± 0.91 ^cd^
T_9_	0.90 ± 0.020 ^b^	1.00 ± 0.00 ^a^	0.93 ± 0.020 ^b^	1527.08 ± 214.58 ^b^	21.66 ± 2.16 ^bc^

The results were recorded as the mean of triplicates ± Standard Error (SE). Different superscript letters indicated the significant differences (*p* ≤ 0.05) (Tukey’s test). Abbreviations: T_1_, Dist water (control); T_2_, spore suspension of *F. oxysporum*; T_3_, silver nitrate solution 1 mM; T_4_, (10 mg/L of AgNPs); T_5_, (20 mg/L of AgNPs); T_6_, (30 mg/L of AgNPs); T_7_, (10 mg/L of AgNPs and spore suspension of *F. oxysporum*); T_8_, (20 mg/L of AgNPs and spore suspension of *F. oxysporum*); T_9_, (30 mg/L of AgNPs and spore suspension of *F. oxysporum*).

**Table 7:** The effects of various treatments on mitotic indices (MI%), phase indices (PI%), types and total abnormalities (Tab%) in *H. vulgare* root tips.

Treatment	MI%	Phase Index (PI%)								Total Abnormal (Tab%)
		Prophase %		Metaphase %		Anaphase %		Telophase %		
		Mitotic	Abn.	Mitotic	Abn.	Mitotic	Abn.	Mitotic	Abn.	
T_1_	10.89 ± 0.65	44.69 ± 2.36	ND	32.87 ± 2.55	3.68 ± 1.06	9.57 ± 1.75	0.92 ± 0.50	12.87 ± 1.79	3.16 ± 1.07	7.76 ± 1.48
T_2_	9.61 ± 0.43 **	33.75 ± 2.38	ND	37.78 ± 2.54	13.69 ± 1.96	6.81 ± 1.76	3.60 ± 1.46	21.66 ± 2.20	6.40 ± 1.37	23.68 **** ± 1.36
T_3_	11.33 ± 0.51 ^ns^	34.72 ± 1.86	ND	36.74 ± 2.28	12.74 ± 1.82	7.71 ± 1.64	3.77 ± 0.89	20.83 ± 2.45	3.86 ± 1.10	20.37 **** ± 1.21
T_4_	10.81 ± 0.45 ^ns^	29.33 ± 1.89	ND	43.43 ± 2.69	6.48 ± 1.47	5.86 ± 1.46	1.78 ± 0.82	21.15 ± 2.55	3.12 ± 1.15	11.38 * ± 1.64
T_5_	13.16 ± 0.56 ***	38.11 ± 2.54	ND	35.90 ± 2.98	9.95 ± 1.49	5.00 ± 1.24	1.71 ± 0.70	20.99 ± 1.97	5.76 ± 1.13	17.42 **** ± 1.45
T_6_	9.95 ± 0.42 *	37.30 ± 2.25	ND	32.28 ± 2.91	9.13 ± 1.85	5.04 ± 1.32	2.81 ± 1.00	25.38 ± 2.54	6.57 ± 1.52	18.51 **** ± 1.55
T_7_	13.95 ± 0.55 ****	34.11 ± 2.33	ND	33.44 ± 2.83	7.50 ± 1.65	8.15 ± 1.83	2.22 ± 0.92	24.30 ± 2.98	6.94 ± 1.86	16.66 *** ± 2.41
T_8_	11.45 ± 0.83 ^ns^	30.67 ± 3.43	ND	30.89 ± 3.67	13.51 ± 3.01	21.70 ± 4.31	4.17 ± 1.56	18.97 ± 2.31	3.56 ± 1.06	21.25 **** ± 2.77
T_9_	13.42 ± 0.61 ***	35.24 ± 2.62	ND	30.58 ± 2.63	6.90 ± 1.59	10.89 ± 2.59	4.72 ± 1.72	23.29 ± 3.40	6.28 ± 2.59	17.90 *** ± 2.63

The results were recorded as the mean of triplicates ± standard error (SE). Total number of examined cells = 2000, ND = not detectable, ns = not significant at 0.05 level from control, *, **, ***, **** indicated significance levels at *p* ≤ 0.01, 0.002, 0.0005, and 0.0001, respectively. T_1_—Distilled water (control), T_2_—spore suspension of *F. oxysporum*, T_3_—AgNO_3_ solution 1 mM, T_4_—(10 mg/L of AgNPs), T_5_—(20 mg/L of AgNPs), T_6_—(30 mg/L of AgNPs), T_7_—(10 mg/L of AgNPs and spore suspension of *F. oxysporum*), T_8_—(20 mg/L of AgNPs and spore suspension *of F. oxysporum*), T_9_—(30 mg/L of AgNPs and spore suspension of *F. oxysporum*).

**Table 8:** Types of chromosomal abnormalities and their respective percentages in each mitotic phase of *H. vulgare* root tips subjected to various treatments.

Treatments	Metaphase					Anaphase						Telophase				
	Stickiness	Non-Congression	Oblique	Disturbed	chr. ring	Bridge	Late Separation	Laggard	Diagonal	Oblique	Disturbed	Bridge	Late Separation	Diagonal	Oblique	Disturbed
T_1_	0.88	0.00	0.00	2.58	0.22	0.00	0.20	0.00	0.39	0.33	0.00	0.00	0.00	0.85	2.31	0.00
T_2_	8.67	1.35	0.00	1.24	2.43	0.00	3.02	0.00	0.23	0.35	0.00	0.35	1.67	2.43	1.69	0.26
T_3_	4.42	1.02	0.29	3.74	3.27	1.04	1.29	0.00	1.18	0.00	0.26	0.00	0.41	2.54	0.41	0.51
T_4_	4.10	0.70	0.29	1.39	0.00	0.00	0.56	0.00	0.22	1.00	0.00	0.00	0.40	2.72	0.00	0.00
T_5_	3.07	1.10	0.33	4.72	0.72	0.29	0.22	0.00	1.00	0.00	0.20	0.00	0.79	2.76	2.20	0.00
T_6_	3.07	0.56	0.00	4.50	1.00	0.00	1.84	0.29	0.69	0.00	0.00	0.00	1.22	4.94	0.40	0.00
T_7_	5.54	0.56	0.00	0.90	0.51	0.00	0.65	0.40	0.00	0.33	0.83	0.00	0.25	4.64	1.56	0.50
T_8_	5.34	0.76	0.00	5.61	1.80	0.00	1.74	0.00	1.99	0.44	0.00	0.00	1.38	0.97	1.21	0.00
T_9_	2.57	0.81	0.00	3.27	0.25	0.00	1.93	1.11	0.72	0.00	0.96	0.32	2.41	3.18	0.00	0.37

## Data Availability

All data generated or analyzed during this study are included in this published Article.
